# Supplementary material for: Effects of a clinical decision support system and patient portal for preventing medication-related falls in older fallers: Protocol of a cluster randomized controlled trial with embedded process and economic evaluations (ADFICE_IT)
Source: PLoS One. 2023 Sep 26;18(9):e0289385. doi: 10.1371/journal.pone.0289385 (PMC10522018; doi:10.1371/journal.pone.0289385)
Supplement: S2 File — (DOCX) [file pone.0289385.s003.docx]

**Items from the World Health Organization Trial Registration Data Set**

1. **Primary Registry and Trial Identifying Number**
   Name of Primary Registry, and the unique ID number assigned by the Primary Registry to this trial.

Name of Primary Registry: ClinicalTrials.gov

ID number assigned by the Primary Registry to this trial: NCT05449470

1. **Date of Registration in Primary Registry**
   Date when trial was officially registered in the Primary Registry.

7-7-2022

1. **Secondary Identifying Numbers**
   Other identifiers besides the Trial Identifying Number allocated by the Primary Registry, if any. These include:
   - The Universal Trial Number (UTN)
   - Identifiers assigned by the sponsor (record Sponsor name and Sponsor-issued trial number (e.g. protocol number))
   - Other trial registration numbers issued by other Registries (both Primary and Partner Registries in the WHO Registry Network, and other registries)
   - Identifiers issued by funding bodies, collaborative research groups, regulatory authorities, ethics committees / institutional review boards, etc.

All secondary identifiers will have 2 elements: an identifier for the issuing authority (e.g. NCT, ISRCTN, ACTRN) plus a number.

There is no limit to the number of secondary identifiers that can be provided.

Identifier as given by the Dutch Central Committee on Research Involving Human Subjects (<https://english.ccmo.nl/>): NL76386.018.21

Identifier as given by the Medical Ethics review board of the Amsterdam University Medical Centres: METC AMC 2021_061

1. **Source(s) of Monetary or Material Support**
   Major source(s) of monetary or material support for the trial (e.g. funding agency, foundation, company, institution).

The ADFICE_IT study is supported by funding from the Netherlands Organization for Health Research and Development (ZonMw, Grant 848017004), The Hague and the Amsterdams Universiteitsfonds: Gepersonaliseerde Medicatieaanpassing bij Oudere Vallers.

1. **Primary Sponsor**
   The individual, organization, group or other legal entity which takes responsibility for initiating, managing and/or financing a study. The Primary Sponsor is responsible for ensuring that the trial is properly registered. The Primary Sponsor may or may not be the main funder.

The sponsor of this study is the Amsterdam UMC.

1. **Secondary Sponsor(s)**
   Additional individuals, organizations or other legal persons, if any, that have agreed with the primary sponsor to take on responsibilities of sponsorship.

   A secondary sponsor may have agreed to:
   - take on all the responsibilities of sponsorship jointly with the primary sponsor; or
   - form a group with the Primary Sponsor in which the responsibilities of sponsorship are allocated among the members of the group; or
   - act as the Primary Sponsor’s legal representative in relation to some or all of the trial sites.

There are no secondary sponsors.

1. **Contact for Public Queries**
   Email address, telephone number and postal address of the contact who will respond to general queries, including information about current recruitment status.

   “Note: The information provided in here is functional and not personal, it is recommended to provide institutional and not personal information. By providing this information the registrant consents that the information provided can or may be published on a public website. Once provided the information cannot be redacted or anonymized as a result of new privacy legislation such as the European General Data Protection Regulation (GDPR)”.

Contact: N. van der Velde

Telephone number: +3120 566 9111

Address: Amsterdam UMC location University of Amsterdam, Internal Medicine, Section of Geriatric Medicine, Meibergdreef 9, 1105 AZ Amsterdam, Netherlands

1. **Contact for Scientific Queries**
   There must be clearly assigned responsibility for scientific leadership to a named Principal Investigator. The PI may delegate responsibility for dealing with scientific enquiries to a scientific contact for the trial. This scientific contact will be listed in addition to the PI.

   “Note: The information provided in here is functional and not personal, it is recommended to provide institutional and not personal information. By providing this information the registrant consents that the information provided can or may be published on a public website. Once provided the information cannot be redacted or anonymized as a result of new privacy legislation such as the European General Data Protection Regulation (GDPR)”.

   The contact for scientific queries must include:
   - Name and title, email address, telephone number, postal address and affiliation of the Principal Investigator, and;
   - Email address, telephone number, postal address and affiliation of the contact for scientific queries about the trial (if applicable). The details for the scientific contact may be generic (that is, there does not need to be a named individual): e.g. a generic email address for research team members qualified to answer scientific queries.

Contact: Prof. Dr. Nathalie van der Velde

Telephone number: +3120 566 9111

Address: Amsterdam UMC location University of Amsterdam, Internal Medicine, Section of Geriatric Medicine, Meibergdreef 9, 1105 AZ Amsterdam, Netherlands

1. **Public Title**
   Title intended for the lay public in easily understood language.

A Clinical Decision Support System and Patient Portal for Preventing Medication-related Falls in Older Patients

1. **Scientific Title**
   Scientific title of the study as it appears in the protocol submitted for funding and ethical review. Include trial acronym if available.

A Clinical Decision Support System and Patient Portal for Preventing Medication-related Falls in Older Patients (ADFICE_IT)

1. **Countries of Recruitment**
   The countries from which participants will be, are intended to be, or have been recruited at the time of registration.

Netherlands

1. **Health Condition(s) or Problem(s) Studied**
   Primary health condition(s) or problem(s) studied (e.g., depression, breast cancer, medication error).

   If the study is conducted in healthy human volunteers belonging to the target population of the intervention (e.g. preventive or screening interventions), enter the particular health condition(s) or problem(s) being prevented.

Prevention of medication-related injurious falls.

1. **Intervention(s)**
   For each arm of the trial record a brief intervention name plus an intervention description.

   Intervention Name: For drugs use generic name; for other types of interventions provide a brief descriptive name.
   - For investigational new drugs that do not yet have a generic name, a chemical name, company code or serial number may be used on a temporary basis. As soon as the generic name has been established, update the associated registered records accordingly.
   - For non-drug intervention types, provide an intervention name with sufficient detail so that it can be distinguished from other similar interventions.

Intervention Description: Must be sufficiently detailed for it to be possible to distinguish between the arms of a study (e.g. comparison of different dosages of drug) and/or among similar interventions (e.g. comparison of multiple implantable cardiac defibrillators). For example, interventions involving drugs may include dosage form, dosage, frequency and duration.

If the intervention is one or more drugs then use the International Non-Proprietary Name for each drug if possible (not brand/trade names). For an unregistered drug, the generic name, chemical name, or company serial number is acceptable.

If the intervention consists of several separate treatments, list them all in one line separated by commas (e.g. "low-fat diet, exercise").

For controlled trials, the identity of the control arm should be clear. The control intervention(s) is/are the interventions against which the study intervention is evaluated (e.g. placebo, no treatment, active control). If an active control is used, be sure to enter in the name(s) of that intervention, or enter "placebo" or "no treatment" as applicable. For each intervention, describe other intervention details as applicable (dose, duration, mode of administration, etc).

Intervention Name: AD*F*ICE_IT CDSS and Patient Portal for optimizing deprescribing of fall-risk-increasing drugs

In this study we will evaluate the effect of an intervention comprised of the combined use of a clinical decision support system and a patient portal for optimizing the deprescribing of FRIDs in older fallers. Patients in the control arm will receive care-as-usual.

1. **Key Inclusion and Exclusion Criteria**
   Inclusion and exclusion criteria for participant selection, including age and sex. Other selection criteria may relate to clinical diagnosis and co-morbid conditions; exclusion criteria are often used to ensure patient safety.

   If the study is conducted in healthy human volunteers not belonging to the target population (e.g. a preliminary safety study), enter "healthy human volunteer".

Patients meeting the following criteria are eligible for inclusion:

- Aged 65 years and older;
- History of at least one fall in the past year;
- A Mini-Mental State Examination (MMSE) score of 21 points or higher or equivalently a Montreal Cognitive Assessment (MOCA) Dutch score of 16 points or higher [50];
- Use of at least one FRID (as defined by the Dutch Federation of Medical Specialists [51]);
- Sufficient command of the Dutch language in speech and writing; and
- Willingness to sign informed consent.

Potential subjects will be excluded if they:

- Already participate in another (intervention) study;
- Have a life expectancy of less than one year; or
- Suffer from severe mobility impairment (i.e. bedridden, e.g. inability to walk short distances with assistance of a walking aid).

1. **Study Type**
   Study type consists of:
   - Type of study (interventional or observational)
   - Study design including:
     - Method of allocation (randomized/non-randomized)
     - Masking (is masking used and, if so, who is masked)
     - Assignment (single arm, parallel, crossover or factorial)
     - Purpose
   - Phase (if applicable)

For randomized trials: the allocation concealment mechanism and sequence generation will be documented.

This study is a a multicenter, cluster-randomized controlled trial. Masking will not be used. Assignment will be at random and at the level of the cluster.

1. **Date of First Enrollment**
   Anticipated or actual date of enrolment of the first participant.

The first patient was enrolled on 7 July 2022.

1. **Sample Size**
   Sample Size consists of:
   - Number of participants that the trial plans to enrol in total.
   - Number of participants that the trial has enrolled.

The trial plans on enrolling 856 participants. A total of 8 participants have currently been enrolled at the point of this submission.

1. **Recruitment Status**
   Recruitment status of this trial:
   - Pending: participants are not yet being recruited or enrolled at any site
   - Recruiting: participants are currently being recruited and enrolled
   - Suspended: there is a temporary halt in recruitment and enrolment
   - Complete: participants are no longer being recruited or enrolled
   - Other

The status of this trial is: recruiting (participants are currently being recruited and enrolled).

1. **Primary Outcome(s)**
   Outcomes are events, variables, or experiences that are measured because it is believed that they may be influenced by the intervention.

   The Primary Outcome should be the outcome used in sample size calculations, or the main outcome(s) used to determine the effects of the intervention(s). Most trials should have only one primary outcome.

   For each primary outcome provide:
   - The name of the outcome (do not use abbreviations)
   - The metric or method of measurement used (be as specific as possible)
   - The timepoint(s) of primary interest

Example:
Outcome Name: Depression
Metric/method of measurement: Beck Depression Score
Timepoint: 18 weeks following end of treatment

The primary outcome is time to first injurious fall. Injurious falls will be recorded prospectively over a period of one year, using weekly fall calendars.

1. **Key Secondary Outcomes**
   Secondary outcomes are outcomes which are of secondary interest or that are measured at timepoints of secondary interest. A secondary outcome may involve the same event, variable, or experience as the primary outcome, but measured at timepoints other than those of primary interest.

   As for primary outcomes, for each secondary outcome provide:
   - The name of the outcome (do not use abbreviations)
   - The metric or method of measurement used (be as specific as possible)
   - The timepoint(s) of interest

Number of injurious falls [ Time Frame: 12 months ]

This concerns the total number of injuirous falls over the course of 12 months. An injurious fall is defined as a fall resulting in wounds, bruises, sprains, cuts, medically recorded fractures, head or internal injury, requiring medical/health professional examination, accident and emergency treatment, or inpatient treatment.

Total number of falls [ Time Frame: 12 months ]

Total number of any fall (I.e. a fall that results in no injuries, or minor, moderate, or severe injuries)

Time to first fall resulting in any injuries [ Time Frame: 12 months ]

I.e. a fall that results in minor, moderate, or severe injuries

Total number of falls resulting in any injuries [ Time Frame: 12 months ]

I.e. a fall that results in minor, moderate, or severe injuries

Time to first non-injurious fall [ Time Frame: 12 months ]

I.e. a fall that results in no injuries

EuroQol-5D-5L (EQ-5D-5L) [ Time Frame: at baseline, 3 months, 6 months, and 12 months ]

The descriptive system comprises five dimensions: mobility, self-care, usual activities, pain/discomfort and anxiety/depression. Each dimension in the EQ-5D-5L has five response levels: no problems (Level 1); slight; moderate; severe; and extreme problems (Level 5). Furthermore, it includes a visual analogue scale (EQ-VAS) which provides a single global rating of self-perceived health and is scored on a 0 (worst health imaginable) to 100 (best health imaginable) scale.

The Older Persons and Informal Caregivers Minimum Data Set-Short Form (TOPICS-SF) [ Time Frame: at baseline and 12 months ]

Data as measured by the The Older Persons and Informal Caregivers Minimum Data Set-Short Form (TOPICS-SF) will be analysed based on the preference-weighted score, ranging from 1.90 to 9.78, with higher scores reflecting a better health status, as perceived by the respondent. The TOPICS - Short Form 2017 including Casemix forms were developed in collaboration with the Nederlandse Vereniging voor Klinische Geriatrie (NvKG - Dutch Association for Clinical Geriatrics) to use as a Patient Reported Outcome Measure (PROM) in the Dutch outpatient and clinical daily practice.

iMTA Productivity Cost Questionnaire (iPCQ) [ Time Frame: at baseline, 3 months, 6 months, and 12 months ]

Direct and indirect costs related to the intervention and care as usual will be assessed using the iMTA Productivity Cost Questionnaire (iPCQ).

iMTA Medical Consumption Questionnaire (iMCQ) [ Time Frame: at baseline, 3 months, 6 months, and 12 months ]

The iMTA Medical Consumption Questionnaire (iMCQ) is an instrument for measuring medical consumption. The iMCQ includes questions related to frequently occurring contacts with health care providers.

Feasibility assessed by number of CDSS and patient portal use [ Time Frame: 12 months ]

To assess the feasibility of the intervention, the investigators will use data logged by the CDSS and patient portal to understand how (often) the CDSS and patient portal are used

Percentage of physicians attending the CDSS training via a questionnaire [ Time Frame: 12 months ]

To assess the feasibility of the intervention, the investigators will look at the percentage of physicians who attended the CDSS training. More specifically, this will be measured by asking physicians whether they attended the CDSS training online, offline or not at all as part of the CDSS user satisfaction questionnaire.

Correlation of percentage of physicians attending the CDSS training and CDSS user satisfaction [ Time Frame: 12 months ]

The correlation between the proportion of a department's staff members who did/did not participate in the CDSS training and user satisfaction regarding the CDSS will be assessed.

CDSS user satisfaction [ Time Frame: 12 months ]

To assess the feasibility of the intervention, the investigators will study the satisfaction regarding the CDSS (i.e. physician evaluations of the CDSS). Agreement with satisfaction statements will be scored on a 7-point Likert scale (1= totally disagree; 7 = totally agree).

Technology Acceptance Model (TAM) [ Time Frame: at baseline ]

The Technology Acceptance Model (TAM) is designed to measure the adoption of a new technology/system based on user attitudes. 6 items aim to measure Perceived Usefulness on a 7-point Likert scale (1=totally disagree; 7 = totally agree), and 6 items aim to measure Perceived Ease of Use on a 7-point Likert scale (1=totally disagree; 7 = totally agree). Intention to use is measured through 1 item on a 7-point Likert scale (1=totally disagree; 7 = totally agree)

Website Satisfaction Scale (WSS) [ Time Frame: at 3 months ]

The Website Satisfaction Scale (WSS) measures satisfaction with comprehensibility, satisfaction with attractiveness, and satisfaction with emotional support through 12 items, for each sub scale using a 7-point Likert response scale, ranging from 1 'totally disagree' to 7 'totally agree'.

Observer OPTION Multiple Chronic Conditions (OPTION-MCC) [ Time Frame: 12 months ]

Videotaped consultations will be coded on triadic decision making in older patients with multiple chronic conditions by using the Observer OPTION Multiple Chronic Conditions (OPTION-MCC) coding scheme. Six types of physicians', patients', and caregivers' behaviors are coded. Physicians' behavior is coded on a 5-point scale (0= The behavior is not observed; 4=The behavior is executed to a very high standard), patients' behavior is coded on a 3-point scale (0=No or minimal participation, e.g. only yes or no; 2=Active participation, answers questions and asks questions, brings in own ideas and shares perceptions), and informal caregivers' behavior is coded on a 3-point scale (0=No or minimal participation, e.g. only yes or no; 2=Active participation, answers questions and asks questions, brings in own ideas and shares perceptions)

Rate of adherence to new medication plan using pharmacy records [ Time Frame: 12 months ]

To assess adherence to the medication advice, the investigators will compare a patient's new medication advice with their pharmacy records to determine whether a patient adheres to the new medication advice or whether they (eventually) change back to their old medication

Number of falls calendar entries [ Time Frame: 12 months ]

To assess adherence to the medication advice, the investigators will compare the new medication advice with falls calender entries on medication use to determine whether a patient adheres to the new medication advice or whether they (eventually) change back to their old medication

iSHARE [ Time Frame: 12 months ]

To evaluate how the intervention facilitates SDM, the investigators will use the iSHAREpatient and iSHAREphysician questionnaires. The iSHAREphysician consists of 16 items scored on a 6-point Likert scale (1= did not do this at all; 6 = completely did this). The iSHAREpatient consists of 16 items scored on a 6-point Likert scale (1= did not do this at all; 6 = completely did this). Dimension scores (range, 0-5) and a total score (the sum of the dimension scores; range, 0-30) for both iSHARE questionnaires will be calculated. The investigators will then apply a linear transformation to obtain a 0 to 100 total score ((score/30)*100). Higher dimension and total scores indicate higher levels of SDM.

Decisional Conflict Scale (DCS; low literacy scale) [ Time Frame: at baseline ]

To evaluate how the intervention facilitates SDM, the investigators will use the Decisional Conflict Scale (DCS; low literacy scale). This scale consists of 10 questions, scored on 3 response categories (yes, do not know, no).

Preparation for Decision-making scale (PrepDM) [ Time Frame: at baseline ]

To evaluate how the intervention facilitates SDM, the investigators will use the Preparation for Decision-making scale (PrepDM). This scale consists of 10 items, scored on a 5-point Likert scale (1= not at all; 5 = a great deal)

Netherlands Patient Information Recall Questionnaire (NPIRQ) [ Time Frame: 12 months ]

To evaluate how the intervention facilitates SDM, the investigators will use the Netherlands Patient Information Recall Questionnaire (NPIRQ). This questionnaire consists of open questions.

1. **Ethics Review**
   The ethics review process information of the trial record in the primary register database. It consists of:
   - Status (possible values: Not approved, Approved, Not Available)
   - Date of approval
   - Name and contact details of Ethics committee(s)

Our study protocol was reviewed and approved by the Medical Ethics review board of the Amsterdam University Medical Centres. The date of approval was: 28/09/2021. The committee can be contacted by mail: [mecamc@amsterdamumc.nl](mailto:mecamc@amsterdamumc.nl)

1. **Completion date**
   Date of study completion: The date on which the final data for a clinical study were collected (commonly referred to as, "last subject, last visit").

N/A: data collection is still ongoing.

1. **Summary Results**
   It consists of:
   - Date of posting of results summaries
   - Date of the first journal publication of results
   - URL hyperlink(s) related to results and publications
   - Baseline Characteristics: Data collected at the beginning of a clinical study for all participants and for each arm or comparison group. These data include demographics, such as age and sex, and study-specific measures.
   - Participant flow: Information to document the progress and numbers of research participants through each stage of a study in a flow diagram or tabular format.
   - Adverse events: An unfavorable change in the health of a participant, including abnormal laboratory findings, and all serious adverse events and deaths that happen during a clinical study or within a certain time period after the study has ended. This change may or may not be caused by the intervention being studied.
   - Outcome measures: A table of data for each primary and secondary outcome measure and their respective measurement of precision (eg a 95% confidence interval) by arm (that is, initial assignment of participants to arms or groups) or comparison group (that is, analysis groups), including the result(s) of scientifically appropriate statistical analyses that were performed on the outcome measure data, if any.
   - URL link to protocol file(s) with version and date
   - Brief summary

Data collection is still ongoing. Results of this study have not yet been published or submitted to any journal.

1. **IPD sharing statement**
   Statement regarding the intended sharing of deidentified individual clinical trial participant-level data (IPD). Should indicate whether or not IPD will be shared, what IPD will be shared, when, by what mechanism, with whom and for what types of analyses. It consists of:
   - Plan to share IPD (Yes, No)
   - Plan description

We plan on sharing the IPD after the trial has been completed, all research data will be made available for other researchers for replication purposes and for original research questions. To obtain data, researchers will need to submit an analysis proposal, which will be evaluated by the steering group.
